# An interesting case of coexistence of autosomal dominant hypocalcemia 1 with Bartter syndrome and chronic myelogenous leukemia

**DOI:** 10.1007/s42000-025-00706-7

**Published:** 2025-08-04

**Authors:** Maria Eleni Chondrogianni, Panagiotis Diamantopoulos, Anna Papadopoulou, Aikaterini Kaperda, Ioannis Kyrou, Harpal S. Randeva, Elpis Vlachopapadopoulou, Maria Yavropoulou, Anna Angelousi, Eva Kassi

**Affiliations:** 1 Division of Diabetes, Endocrinology and Metabolic Diseases, First Department of Propaedeutic and Internal Medicine, National and Kapodistrian University of Athens, General Hospital of Athens “LAIKO”Athens, Athens, Greece; 2 Center of Expertise for Rare Endocrine Diseases (C.E.R.E.D), General Hospital of Athens, LAIKO, National and Kapodistrian University of Athens, Athens, Greece; 3 European Reference Network on Rare Endocrine Conditions (ENDO-ERN) accredited, Athens, Greece; 4 Department of Biological Chemistry, Medical School, National and Kapodistrian University of Athens, Athens, Greece; 5 Department of Internal Medicine, National and Kapodistrian University of Athens, Athens, 1st Greece; 6 Department of Cninical Biochemistry, National and Kapodistrian University of Athens Medical School, University General Hospital “Attikon”, Athens, Greece; 7 Warwickshire Institute for the Study of Diabetes, Endocrinology and Metabolism (WISDEM), University Hospital Coventry & Warwickshire, Coventry, UK; 8 Institute for Cardiometabolic Medicine, University Hospitals Coventry and Warwickshire NHS Trust, Coventry, UK; 9 University of Warwick, Warwick Medical School, Coventry, UK; 10 Centre for Health & Life Sciences, Coventry University, Coventry, UK; 11 Aston Medical School, College of Health and Life Sciences, Aston University, Birmingham, UK; 12 College of Health, Psychology and Social Care, University of Derby, Derby, UK; 13 Department of Endocrinology-Growth and Development, P. & A. Kyriakou Children’s Hospital, Athens, Greece; 14 Unit of Endocrinology1st, Department of Internal Medicine, National and Kapodistrian University of Athens, Athens, Greece

## Abstract

**Backround**: Autosomal dominant hypocalcemia type 1 (ADH1) is a rare form of hypoparathyroidism caused by heterozygous, inherited, or de novo activating mutations in the calcium-sensing receptor gene (CASR).

**Case presentation**: A 29-year-old man was referred to the outpatient department for poorly controlled hypoparathyroidism with hypocalcemia, hypomagnesemia, mild hypokalemia, excessive hypercalciuria, and a worsening eGFR under conventional therapy. He was diagnosed with hypoparathyroidism on the second day of birth. He remained inadequately controlled even after the initiation of replacement therapy with rhPTH (1–84). He was also diagnosed with chronic myelogenous leukemia (CML) at the age of 28 years and was treated with tyrosine kinase inhibitor (TKI), which further worsened the control of hypoparathyroidism. Genetic analysis was performed at the age of 31 years and revealed a change c.2486 A > G in exon 7 of the *CaSR* gene.

**Conclusion**: This case highlights the importance of characterizing the cause of non-surgical hypoparathyroidism, including ADH1, in the differential diagnosis. ADH1 may coexist with Bartter syndrome type V, making the patient’s management more challenging. To our knowledge, this is the second case in the literature with the coexistence of two rare diseases, ADH1 and CML.

In CML patients treated with TKIs, serum calcium levels should be monitored and, in the case of severe hypocalcemia accompanied by low or inappropriately normal PTH, the possible existence of ADH1 may need to be investigated.

## Introduction

Hypoparathyroidism is a metabolic disorder characterized by low calcium and low or inappropriately normal parathyroid hormone (PTH) concentrations [1, 2]. The most common cause of hypoparathyroidism is post-operative (75% of all cases). However, there are also autoimmune or genetic causes of hypoparathyroidism [2]. Autosomal dominant hypocalcemia (ADH) type 1 is caused by heterozygous, inherited or de novo activating mutations in the calcium-sensing receptor gene (CaSR) [3]. It is the most frequent genetic cause of isolated hypoparathyroidism and its prevalence is estimated at 3.9 cases per 100,000 persons [4]. CaSR is a dimeric family C G-protein coupled receptor, expressed in many calcitropic and non-calcitropic tissues, though more abundantly in cells of the parathyroid glands and the renal tubular cells, that binds to extracellular ionized calcium (Ca^2+^) among other ligands. It is a natural sensor of extracellular Ca^2+^ concentration [5]. Elevated extracellular calcium concentration activates the CaSR, leading to augmented intracellular calcium levels, suppression of PTH synthesis and secretion, and enhancement of intracellular degradation of PTH [4]. More than 100 *CASR* gain-of-function variants have been described [6]. Activation of CaSR either due to mutations or ligands results in increased intracellular signaling and thus a decrease in PTH synthesis and secretion [3, 5]. In the kidneys, CaSR activation leads to a decrease in Ca^2+^ reabsorption [3]. Approximately 50% of patients with ADH1 have hypercalciuria [4]. Hypercalciuria is defined as a 24-hour urinary calcium level of > 7.5 mmol/24 hours or > 300 mg/24 hours for adult males and > 6.25 mmol/24 hours or > 250 mg/24 hours for adult females [7], while there is no specific definition for patients with ADH1 [4]. Inactivating mutations in the same gene have the opposite effect, leading to hypercalcemia and hypocalciuria [8].

Bartter syndrome (BS) is a hereditary renal tubular disorder which causes a disorder of salt reabsorption in the thick ascending limb (TAL) of the loop of Henle, resulting in extracellular fluid volume depletion, hypokalemia, metabolic alkalosis, hyperreninaemia, and hyperaldosteronaemia [9]. There are several forms of BS resulting from mutations in genes encoding transporters that play a role in NaCl reabsorption in the thick ascending limb of the loop of Henle [10]. BS type V, also referred to as “ADH with Bartter-like syndrome”, is caused by heterozygous activating mutations in the CASR gene that lead to augmented CaSR activity, which inhibits the Na-K-2Cl cotransporter thus causing the inhibition of NaCl transport in the TAL [11]. The CaSR is also expressed in other noncalcitropic tissues, such as pancreatic islets, lungs, small and large intestine, brain, skin, and vascular system, where it plays a role in regulation of gene expression and cell proliferation, differentiation, and apoptosis [12]. The dysfunction of the CaSR has been associated with several diseases, including some types of cancer [12].

Chronic myeloid leukemia (CML) is a myeloproliferative neoplasm and occurs in three different phases (the chronic, accelerated, and blast phase) [13]. Recently, CaSR has been raised as a targetable factor in AML progression; however, data on its role in CML are scarce [14].

## Aim

The aim is to report on a patient who presented with coexistence of two rare diseases, namely, ADH1 with BS type V and CML, which potentially may be interrelated to and reinforced by one another pathophysiologically and clinically.

## Case presentation

A 29-year-old man was referred to the outpatient department with hypocalcemia due to poorly controlled hypoparathyroidism along with hypomagnesemia, mild hypokalemia, and worsening eGFR. Hypoparathyroidism was diagnosed on the second day of birth. The patient had a history of nephrolithiasis and basal ganglia calcification. His mother had a history of breast cancer and his father had a history of intramedullary ependymoma. Neither of them reported a history of calcium metabolism disorders. The patient did not report any symptom, probably due to long-standing hypocalcemia, and Chvostek’s and Trousseau’s signs were negative. He was treated with conventional therapy with calcium carbonate (3grs/day divided into four doses), alfacalcidol (2 µg/day), cholecalciferol (1200 IU/ day), and hydroclorothiazide (25 mg/day). Serum calcium was 6.6 mg/dl (reference range: 8.5 to 10.5 mg/dl), serum phosphate level was 6.3 mg/dL (2.7–4.5 mg/dl), serum magnesium was 1.5 mg/dl (1.6–2.4 mg/dL), serum albumin was 4.78 g/dl(3.5–5 g/dl), parathyroid hormone level was < 1.2 pg/mL (12–64 pg/mL), and 24 h urine calcium was 356 mg/24h (< 300 mg/24 h) (Table 1).

**Table 1:**
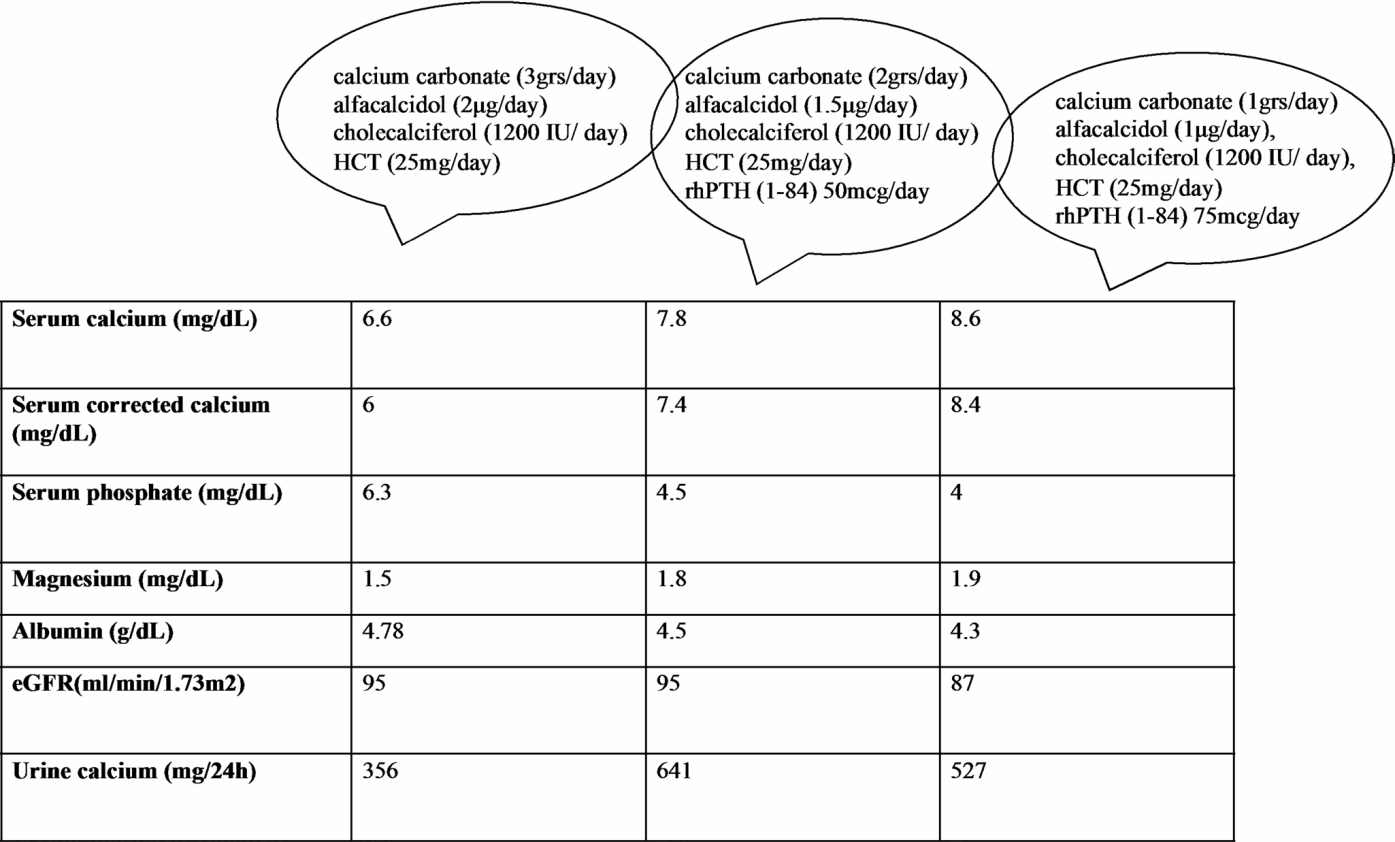
The biochemical profile of the patient before and after the initiation of rhPTH(1–84) treatment. HCT: hydrochlorothiazide, rhPTH: Rrecombinant human parathyroid hormone

In the outpatient department, recombinant parathyroid hormone [rhPTH (1–84)] 50mcg sc. once daily was initiated along with magnesium supplements, leading to a down-titration of the alfacalcidol and calcium supplements and better control of hypocalcemia (Table 1). One year before referral to our department, at the age of 28 years, he presented with leukocytosis, thrombocytosis, and splenomegaly (white blood cell count, 291 × 10^9^/L; platelet count, 582 × 10^9^/L; hemoglobin level, 9.1 g/dl, lactate dehydrogenase level, 1676 U/L, vitamin B12 level, > 4000 pg/ml) and was diagnosed with CML (cytogenetics, 46,XY, t(9;22)(q34.1;q11.2); BCR/ABL1, p210 b3a2). He was initially treated with hydroxyurea at 6 g/day which was replaced by a tyrosine kinase inhibitor (TKI), nilotinib, at 300 mg BID, after a few days (when the result of the molecular testing for BCR/ABL1 became available). Having failed to achieve a major molecular response (MMR) after 12 months on nilotinib (BCR/ABL1 0.75% SI), mutation testing showed the presence of a BCR/ABL1 mutation (F359V) that is resistant to treatment with nilotinib; thus, the treatment was changed to another TKI, dasatinib, at 100 mg QD, enabling the achievement of MMR within the following 3 months. After 3 years on dasatinib, he remains in MMR but without having achieved a complete molecular response.

Of note, the control of hypocalcemia became more difficult after the initation of TKIs. The rhPTH was titrated to 100mcg and various doses of calcium, magnesium, and alfacalcidol supplements along with hydrochlorothiazide were tested with the aim of achieving better management of his hypocalcemia, excessive hypercalciuria, hypokalemia, and hypotension. However, control of the disease remained suboptimal, as is expected in ADH1.

### Genetic analysis

After obtaining the patient’s informed consent, next-generation sequencing (NGS) was performed on a NextSeq2000 platform (Illumina) and the Clinical Exome solution v3 gene panel, Sophia Genetics (CES_v3_hg38) was used. Among the genes included in the panel, the analysis was focused on those involved in the development of hypoparathyroidism (*CLCNKB*,* TBCE*,* GATA3*,* JMJD1C*,* CYP2R1*,* HBB*,* PTH*,* TCIRG1*,* CACNA1C*,* CYP27B1*,* FGF23*,* ORAI1*,* VDR*,* ATP7B*,* LIG4*,* TNFSF11*,* NKX2-1*,* UBR1*,* CLCN7*,* IRX5*,* PRMT7*,* SLC12A3*,* ACADVL*,* CTNS*,* PRKAR1A*,* SLC4A1*,* TBX2*,* CCBE1*,* TNFRSF11A*,* RYR1*,* CAMKMT*,* HADHA*,* HADHB*,* LPIN1*,* PPM1B*,* PREPL*,* SLC3A1*,* GNAS*,* PIGT*,* STX16*,* AIRE*,* ALG12*,* COMT*,* DGCR2*,* ESS2*,* GP1BB*,* TBX1*,* UFD1*,* CASR*,* IFT122*,* PTH1R*,* FAT4*,* CSF1R*,* NSUN2*,* PDE4D*,* GCM2*,* OSTM1*,* CAV1*,* CYP3A4*,* SEMA3E*,* SNX10*,* CA2*,* CHD7*,* TRPM6*,* BTK*,* COL4A5*,* and FOXP3*). The analysis revealed the genetic change c.2486 A > G in exon 7 of the *CaSR* gene, which would result in the substitution of the aminoacid tyrosine for cystein at position 829 of the protein (p.Tyr829Cys).

## Discussion

Patients with ADH1 have high phenotypic heterogeneity, making their management challenging [15, 4, 6]. Roszko et al. in their systematic review demonstrated that while 27% of patients with ADH1 were asymptomatic, more than one-third presented with at least one severe symptom, such as seizure, loss of consciousness, and/or laryngospasm [6]. Moreover, they showed that 75% had at least one complication while they were on treatment. They are usually treated as “common” primary hypoparathyroidism with conventional therapy οr PTH analogs. This usually results in aggravation of hypercalciuria [15]. Although calcilytics is probably the most appropriate treatment for ADH1, it is not yet approved. Thus, access is limited and only in clinical trials [16]. PTH replacement therapy has been used in patients with ADH1, either in the form of rhPTH(1–34) (bid, sbc infusion), rhPTH (1–84), or in the form of trancon-PTH [4]. However, in the REPLACE trial, which tested the efficacy and safety of rhPTH (1–84) in hypoparathyroidism, patients with ADH1 were excluded [4, 17]. Winer et al. demonstrated that teriparatide injected twice daily diminished urine calcium excretion while keeping serum calcium below the normal level [18]. Sastre et al. evaluated the effectiveness of continuous sc. rhPTH (1–34) infusion (CSPI) in six young patients with ADH1 and hypocalcemic seizures and concluded that CSPI increases serum calcium and reduces hospital admissions in such patients, while in half of them it reduces calciuria [19]. In another study, by Robert et al., when the calcilytic drug NPSP795, a negative allosteric modulator of the extracellular CaSR, was administered in five patients with ADH1, they achieved better control of serum and urine calcium levels [15].

In our patient, BS coexisted with ADH1, while CML presented later in his life. Two cases with the specific mutation in *CaSR* have been reported up to now. Choi et al. reported a case of a female patient with the same activating mutation in the *CaSR* (p.Tyr829Cys) [11]; in this case, a mild BS type V also coexisted with ADH1. The symptoms occurred on the 12th day after birth. Moreover, nephrocalcinosis and basal ganglia calcification presented at the age of 7. Of interest, the symptoms of BS were intensified during hypercalcemic episodes. The second case of a young patient with ADH1 with the same mutation was reported by Sastre et al. [19].

The clinical presentations and onset timing of BS phenotype differ according to the type of mutation and even between patients who have the same *CaSR* mutation, exacerbating the loss of water and predisposing to hypokalemia to varying extent [20]. Moreover, it was shown that the CaSR activity differs according to the type of mutation [20]. In patients with activating mutations of *CaSR* (K47N and P221L), clinical features of BS were not found [20]. However, patients with C131W and A843E mutations demonstrated characteristics of BS. Vezzoli et al. reported two monozygotic twin sisters with ADH1 who had an activating mutation in the *CaSR* gene (K29E) and they both manifested a mild Bartter-like syndrome at the age of 22 years old [21]. Only one needed potassium supplements for the correction of hypokalemia [21].

Of interest, the present case is the second in the literature with coexistence of ADH1 and CML. Jones-Ryan et al. reported a case of a 69-year old man with CML who had asymptomatic mild hypocalcemia and the initiation of imatinib triggered severe hypocalcemia. Genetic testing revealed a mutation in the CaSR gene (p.Thr151Met) compatible with ADH1 [22].

Moreover, Petric et al. presented a case report of a 61-year-old woman with CML, treated initially with imatinib and then with nilotinib, who was hospitalized due to various side effects, severe hypocalcemia, and ECG changes. The mechanism of hypocalcemia was not completely understood. It was proposed that changes in intestinal and renal calcium handling, bone remodeling, immune-mediated destruction of parathyroid glands or the interaction of the drug with the CaSR could cause hypocalcemia. They also noted the importance of measuring calcium levels during the treatment with such drugs. However, genetic testing was not performed. As a result, the coexistence of ADH1 could not be excluded [23]. Hypocalcemia is also reported as a not well-known and under-recognized side effect of TKIs, occurring in less than 10% of patients [24]. The symptoms being nonspecific, they are often misinterpreted as constituting part of the underlying condition.

The bone marrow microenvironment (BMM) consists of cellular and non-cellular components that interact with the hematopoietic stem cells (HSCs) to achieve a delicate balance between hibernation, self-renewal, and maturation of the hematopoietic precursors [25]. Apart from the numerous genetic, epigenetic, and cellular events affecting the HSCs that may lead to clonal disorders and eventually leukemia, studies on the BMM have shown that disorders of the latter may be implicated, either causally or as a sequela, in the emergence and evolution of leukemia. It has been shown that the localization of HSCs during development is significantly affected by local calcium concentrations [26]. HSCs sense extracellular calcium via CaSR, a ubiquitously present protein that guides HSC adhesion to extracellular matrix proteins. There is also evidence that the extracellular calcium concentration differs between leukemias, while the same applies to the response of different leukemia cells to extracellular calcium concentrations [14]. Of interest, it has been shown that activation of CaSR in other cell types permits calcium to enter the cells via receptor-operated channels (ROCs), causing a CaSR-mediated rise of the intracellular calcium levels [27]. This increase can activate protein kinase C. The calcium-dependent protein kinase (PKC) superfamily, which is a group of serine/threonine-kinase isoforms that are responsible for the modification of a wide range of proteins (receptors, enzymes, and transcription factors) [28], has been found to be involved in cellular processes such as proliferation, differentiation, and survival, thus playing a role in the emergence and progression of malignancies [29]. PKCs have been found to play important roles in survival and prognosis in several hematological malignancies, such as acute myelogenous leukemia, chronic lymphocytic leukemia, hairy cell leukemia, and other lymphoproliferative disorders.

Even for disorders, such as CML, that have been causally characterized, the microenvironment effects may be crucial in the generation and progression of clonality. The hallmark of CML is the fusion of the BCR and ABL1 genes into a chimeric gene (BCR-ABL1) that encodes a protein with constitutive tyrosine-kinase activity, resulting in the constitutive activation of several downstream pathways, responsible for the induction of cellular proliferation, loss of adhesion, and inhibition of apoptosis, eventually leading to the clinical phenotype of CML. Targeting this fusion protein with TKIs has revolutionized the management of CML during the last 25 years. Until recently, no correlation between PKC and CML had been evidenced, although several studies have implied that both PKC- and BCR-ABL1-mediated signaling pathways could lead to similar cellular phenotypes [30]. Erythrocytes from patients with CML have high PKC levels and PKC activity has also been found to be increased in these patients [30]. Finally, PKC isoforms are involved in BCR-ABL1 downstream pathways [30], leading to increased expression of moleculars such as MCL-1 BCL-XL, COX-2 that are essential for the phenotypic changes of CML cells. In this context, an activating mutation of the *CaSR* may result in increased activation of PKC in the BMM, thus contributing to the CML phenotype or even to resistance to TKIs.

In conclusion, in CML patients treated with TKIs and presenting with severe hypocalcemia, the possible existence of ADH1 may need to be investigated. Moreover a potential role of *CaSR*-activating mutations in the pathogenesis and progress of CML merits investigation.

### Learning points

- Autosomal dominant hypocalcemia should be considered in the work-up of hypocalcemia-hypoparathyroidism.
- ADH1 may coexist with Bartter syndrome.
- The management of patients with ADH1 is challenging: PTH analogs may constitute a partial solution but not the optimal treatment for ADH1.
- In CML patients treated with TKIs, calcium levels should be monitored and, in the case of severe hypocalcemia, the possible existence of ADH1 may need to be investigated.
